# Evaluation of non-carious cervical restorations with different cavity configurations: a microscopy and OCT analysis

**DOI:** 10.2340/biid.v13.46121

**Published:** 2026-06-22

**Authors:** Ayla Macyelle de Oliveira Correia, Peter Hamilton Tomlins, Robert William Read, Taciana Marco Ferraz Caneppele, Eduardo Bresciani, Ana Raquel Benetti

**Affiliations:** a Department of Restorative Dentistry, São Paulo State University (UNESP), Institute of Science and Technology, São José dos Campos - SP, Brazil; b Bart’s and the London School of Medicine and Dentistry, Queen Mary University of London (QMUL), London, UK; c Department of Civil and Mechanical Engineering, Technical University of Denmark (DTU), Kongens Lyngby, Denmark; d School of Dentistry and Medical Sciences, Centre for Rural Dentistry and Oral Health, Charles Sturt University (CSU), Orange, Australia; e Department of Odontology, Faculty of Health and Medical Sciences, University of Copenhagen (KU), Copenhagen, Denmark

**Keywords:** Composite resins, non-carious cervical lesions, dental marginal adaptation, optical coherence tomography

## Abstract

The aim of this *in vitro* study was to assess marginal and internal gap formation in simulated non-carious cervical lesions (NCCLs) of different cavity configurations and restored with either bulk-fill or nanofilled resin composite, before and after thermocycling. NCCLs with 2.3 mm diameter and approximately 3.5 mm^3^ volume were prepared in 40 human molars in two cavity configurations (*n* = 20): saucer (1.3 mm depth) or wedge (1.9 mm depth) shape. The adhesive Clearfil SE Bond was used in all groups. These cavities were randomly divided according to the resin composite used (*n* = 10): Filtek Z350 XT or Filtek One Bulk Fill Restorative. The marginal and internal adaptation of the composite at cavity margins was assessed before and after thermal aging (TA) (5000 cycles between 5 and 55°C) using a plane light microscope and optical coherence tomography (OCT), respectively. The 3D images captured by OCT were analyzed by obtaining 16 B-scans per specimen using a custom-made script for software MATLAB R2019a, Update 3. Data were analyzed by two-way analysis of variance and paired *t*-tests (α = 0.05). Regarding the marginal gap, there was no statistically significant difference between the saucer and wedge cavity configurations, before or after TA, nor between cavities restored with bulk-fill or nanofilled composite (*p* > 0.05). Likewise, for the internal gap, no significant differences were found for the cavity configuration nor the resin composite (*p* > 0.05). Restorations placed in saucer or wedge cavity configurations showed similar marginal and internal adaptation, regardless of the type of resin composite, and gap formation was not aggravated by thermocycling.

## Introduction

Non-carious cervical lesions (NCCLs) may present reduced adhesion to composites either due to the presence of sclerotic dentine, the lack of enamel available for bonding at the gingival margins, and/or very particular morphologies deemed unfavorable for retention of restorations [1, 2]. These morphologies depend on the predominant etiological factor and are commonly described as saucer or wedge cavity configurations [3, 4]. Previous findings show that some cavity configurations may favor stress concentration at the bonded interface more than others [5–7], which might contribute to premature failure of the resin composite restorations.

The so-called bulk-fill composites were developed as an alternative to regular composites in an effort to reduce polymerization shrinkage stress and its undesirable consequences [8–13], with promising results in NCCLs [7, 12, 14, 15]. However, it is not known whether bulk-fill composites have a significant effect on stress relief in different NCCL cavity configurations, nor to what extent the internal and marginal adaptation of the restorations could be improved. While early laboratory studies assessed marginal gap formation in composite restorations using microscopy techniques [16–18], a representative and non-destructive overview of the tooth-restoration interface becomes possible by using either micro-computed tomography [19, 20] or optical coherence tomography (OCT) [17, 21–24]. Several studies using OCT offered valuable mapping of the bonded interface, either by assessing the presence of gaps on localized 2D images [17, 21, 25, 26] or by following the 3D progression of gaps in real time [22, 24]. Most of these studies assessed either cylindrical [21], tapered-cylindrical [17, 22, 26], or hemispherical cavities [25] with enamel at the occlusal margin and dentine at the gingival margin. While the cylindrical or tapered-cylindrical cavities are interesting models for mapping the bonded interface using OCT, these cavity types are rarely made in dental practice. Models using semi-spherical cavities [25, 27], on the other hand, are closer to the clinical reality, but such studies are at present limited. Thus, the present study seeks to bring additional knowledge by using OCT in the assessment of cavity types that are very similar to NCCLs. Additionally, building upon important contributions from earlier OCT studies [17, 21, 22, 24–26], more representative information about the gap formation will be sought by a new approach to the image analysis. Rather than the customary assessment on single 2D images, this novel method compiles and averages images obtained from 3D OCT scans. This *in vitro* study aimed to assess internal and marginal gap formation through microscopy and OCT in simulated NCCLs with different cavity configurations and restored with resin composite recommended either for bulk-fill or incremental technique. A further aim was to assess the extent to which gap formation was influenced by thermocycling. The working hypotheses of this study were that internal and marginal gap formation would be affected by (1) different cavity configurations, (2) different types of resin composites, and (3) thermocycling.

## Materials and Methods

### Tooth selection and cavity preparation

Forty human maxillary and mandibular molars extracted for therapeutic reasons were selected for this *in vitro* study. Based on findings from previous work, which employed the same methodology used here to assess marginal gap formation, significant differences between resin-based materials were identified when groups consisted of 10 samples each [28]. Thus, the same number of samples was adopted for the present study, which served as pilot for the novel analysis of the OCT images. The teeth were collected as anonymous biological material and therefore exempt from approval by the National Ethics Committee (cf. Danish Research Ethics Committee’s Act § 14, paragraph 3). The teeth were stored in 0.5% chloramine solution and rinsed in deionized water prior to use. Teeth with carious lesions, hypoplastic defects or cracks were not included.

**Figure 1:**
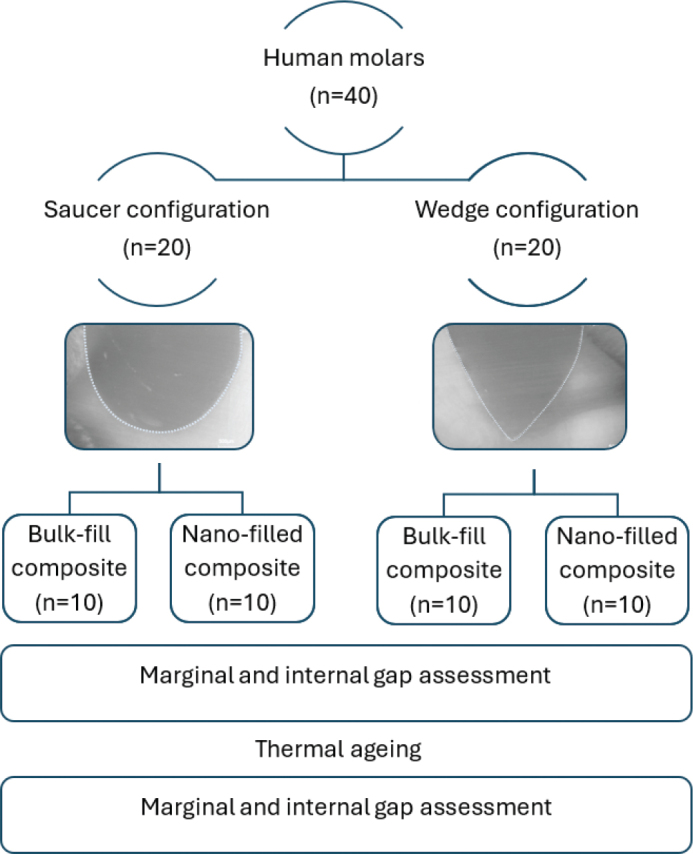
Illustration of study design. Legends: Bulk-fill composite (Filtek™ One Bulk Fill Restorative), Nanofilled composite (Filtek™ Z350 XT). Restorations are outlined in this image to improve visibility.

The teeth were fully embedded in epoxy resin with their buccal surface facing up (EpoFix Resin, Struers, Ballerup, Denmark). After polymerization, the buccal surface of each tooth was carefully exposed and flattened using sequential-grit (no. 80, 200, 500, 1000) silicon carbide abrasive papers (Struers; Ballerup, Denmark) in a polishing device (LaboForce-1, Struers; Ballerup, Denmark), under water irrigation, in order to create a flat surface for standardizing preparations. Flattening of the buccal surface significantly reduced light scattering on the original convex surface of the tooth and improved the OCT image quality needed for assessment of the composite restorations. The experimental design of this study is shown in Figure 1. The specimens were randomly assigned to two groups (*n* = 20) according to the cavity configuration: saucer or wedge. For the saucer configuration (2.3 mm diameter, 1.3 mm depth, and approximately 3.5 mm^3^ volume), the cavities were prepared using spherical diamond burs (801; Komet Dental, Germany). For the wedge configuration (2.3 mm diameter, 1.9 depth, and approximately 3.5 mm^3^ volume), pointed football-shaped diamond burs (368; Komet Dental, Germany) were used to produce ‘V-shaped’ cavities. The burs were attached to a high-speed air turbine handpiece (SIROPure; Sirona, Germany) emitting water spray. The handpiece was then mounted on a flexible robot arm (UR3; Universal Robots, Odense, Denmark) to prepare cavities with standardized dimensions. In order to maintain the cutting efficiency, the bur was changed after five cavity preparations. The occlusal cavity margins were located in enamel, and the gingival margins were in dentine/cementum; all cavity floors were in dentine. The width and the depth of each cavity were confirmed with a commercial swept-source OCT system (IVS‐300, Santec Corporation, Aichi, Japan) using the measuring tool ‘point to point’. After preparation, the cavities were rinsed with deionized water and stored for approximately 1 hour.

### Resin composite restoration

As a continuation from previous work [7, 15], the same adhesive and restorative composites were selected for this study. The saucer and wedge cavities were randomly divided according to the restorative material used (Table 1): nanofilled resin composite recommended for incremental technique (Filtek™ Z350 XT; 3M, St. Paul, MN, USA) or nanofilled resin composite recommended for bulk-fill technique (Filtek™ One Bulk Fill Restorative; 3M, St. Paul, MN, USA). Theoretical work on the same resin composites showed better shrinkage stress distribution at the adhesive interface from the composite recommended for bulk-fill rather than the one for incremental technique [7], thus the potential stress-related effect on the cavity seal was investigated in the present study. The four subgroups (*n* = 10) of each cavity configuration are presented in Figure 1.

**Table 1:** Technical information about adhesive system and resin composites used in this study.

Material	Shade	Lot No.	Manufacturer	Composition
Clearfil™ SE Bond	-	000045	Kuraray America, Inc, New York	Primer: 10-MDP, HEMA, DMA, catalyst, water. Bond: 10-MDP, HEMA, DMA, Bis-GMA, filler, catalyst.
Filtek™ Z350 XT	A2	766910	3M, St. Paul, MN, USA	Filler: 78.5 wt% (59.5 vol%) silica, zirconia, aggregated zirconia/silica. Matrix: Bis-GMA, UDMA, TEGDMA, dimethacrylate.
Filtek™ One Bulk Fill Restorative	A2	N980743	3M, St. Paul, MN, USA	Filler: 76.5 wt% (58.5 vol%) silica, zirconia, ytterbium trifluoride, aggregated zirconia/silica. Matrix: AUDMA, AFM, UDMA, DDDMA.

10-MDP: 10-methacryloyloxydecyl dihydrogen phosphate; HEMA: 2-hydroxyethyl methacrylate; DMA: dimethacrylate; Bis-GMA: bisphenol-glycidyl methacrylate; UDMA: urethanedimethacrylate; TEGDMA: triethylene glycol dimethacrylate; AUDMA: aromatic urethane dimethacrylate; AFM: addition-fragmentation monomer; DDDMA: 1,12-dodecanediol dimethacrylate.

The cavities were restored using a self-etching adhesive system (Clearfil™ SE Bond; Kuraray America). This adhesive was selected because of its documented good long-term clinical performance in NCCLs, also without selective acid etching [29]. First, the primer was gently scrubbed on the cavities for 20 seconds, then a gentle air stream was applied to evaporate the solvent. The cavities were subsequently treated with the bonding agent and cured using a LED curing-light (Elipar™ DeepCure-L LED Curing Light; 3M, St. Paul, MN, USA) for 10 s at 1050 mW/cm^2^, the lamp irradiance confirmed by a radiometer (Bluephase ^®^ Meter II; Ivoclar Vivadent, Austria).

A single increment (bulk placement) was placed in the cavities using one of the above-mentioned resin composites. The composite was covered with a polyester strip, and the curing-light tip was positioned perpendicularly to the cavity and in contact with the polyester strip. Light-curing was done with the LED curing-light (Elipar™ DeepCure-L LED Curing Light; 3M, St. Paul, MN, USA) for 10 seconds. The restoration was then manually ground using 1000-grit silicon carbide abrasive paper (Struers; Ballerup, Denmark) under water irrigation for removal of excess material. Subsequently, the restorations were polished with aluminum oxide suspended in water using a felt disc mounted on a polishing device (LaboForce-1, Struers; Ballerup, Denmark). The restorations were then rinsed with pressurized water to remove surface residues and kept in deionized water for 24 hours at 37°C. The same operator placed all resin composite restorations.

### Marginal gap assessment using light microscopy

Gap formation at enamel and at dentine margins was assessed before and after thermal aging (TA) using a plane light microscope (Ortoplan, Leitz Wetzlar GmbH, Germany) coupled with a digital camera and specific software (DeltaPix InSight, Smorum, Denmark). Immediately before assessment, the surface of the specimens was gently dried using absorbent paper.

Cavity margins were inspected under reflected light using objectives with enlargements of 32x and 64x, and an ocular with enlargement of 8x, i.e., at 256x and 512x magnification, respectively. The two widest gaps identified on the restoration perimeter and their immediately opposite margins were photographed, and two sites at both enamel and dentine margins were measured, aided by the camera’s software (DeltaPix InSight, Smorum, Denmark). An average gap width was calculated for enamel and dentine for all groups. Then, the widest gap and the one directly opposite to it were added, and the total gap was divided by the cavity diameter to calculate the percentage wall-to-wall contraction of the restoration as follows: [(Widest gap + Directly opposite gap)/Cavity diameter] × 100. Thus, marginal gap formation was expressed as wall-to-wall contraction of the composites as a percentage of the cavity’s diameter. This method has previously been used to assess and quantify marginal gaps [30].

### Internal gap assessment using OCT

Gap formation at the cavity’s internal walls was assessed using a commercial swept-source OCT (SS-OCT) system (IVS‐300, Santec Corporation, Aichi, Japan). This system operates with a light source at a wavelength of 1310 ± 30 nm, and the manufacturer reports a resolution of ≤12 μm axially and 22 μm transversally.

The scanning probe of the SS-OCT was placed over the specimen with the light-source beam projected onto the cavity center almost perpendicularly, but at a slight angle to the restoration surface, thus reducing backscattering from the restoration surface. The OCT system was configured to acquire 256 A-Scans in each B-scan and 256 B-scans in a single volume (XYZ volume = 256 × 256 × 968 pixels). Three-dimensional (3D) images centralized at the restoration were obtained for each specimen by scanning the specimen within a surface area of 4 × 4 mm, before and after TA.

### Analysis of the SS-OCT images

As the refractive index of the dental hard tissues is similar to that of the resin composite, the bonded interface cannot be identified when good bonding between the restorative material and the tooth occurs. On the contrary, when air is present between the restoration and the tooth (because of a gap or air entrapment), the light is backscattered, and the intensity of the signal increases. This principle was used to analyze the images acquired with the SS-OCT.

The 3D images were analyzed using a custom-made script for software MATLAB R2019a, Update 3 (The MathWorks Inc., United States). Calibration curves plotting the internal gap length obtained using OCT against microscopy were necessary. Therefore, five specimens from each cavity configuration (saucer and wedge) were centrally sectioned, and the interface between the restoration and the tooth was analyzed using the plane light microscope (Ortoplan, Leitz Wetzlar GmbH, Germany) under 256x and 512x magnification. The sectioned restorations were photographed at 8x magnification to measure the length of the adhesive interface at the sagittal plane. At the optical microscope, the length of the gap identified along the adhesive interface was divided by the total length of the interface to provide the percentage of gap length. In parallel, the corresponding volume in the 3D images obtained using the SS-OCT was analyzed using the custom-made MATLAB script employing different thresholds. The threshold resulting in the highest correlation coefficient between the gap length identified visually using the microscope and the one calculated by software from the 3D OCT images was identified and used in the remaining analyses. The highest correlation coefficients obtained for the saucer and the wedge cavity configurations were, respectively, *R*² = 0.8262 and *R*² = 0.7131, which defined the optimal threshold for the conditions of the present study.

**Figure 2:**
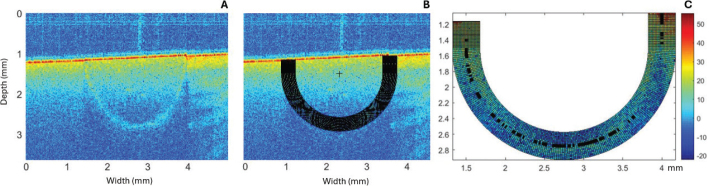
Representation of image analysis for saucer-shaped restorations. (A) Color-mapped average image from 16 B-scans. (B) Average image overlaid by a 20-pixel wide, semi-circular grid at the region of interest. (C) Black pixels show the identifiable interface, i.e. the presence of gap. The percentage of black pixels (gap) in relation to the length of the adhesive interface was calculated in the software.

**Figure 3:**
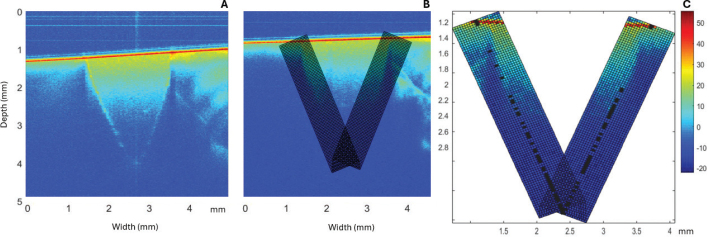
Representation of image analysis for wedge-shaped restorations. (A) Color-mapped average image from 16 B-scans. (B) Average image overlaid by a 20-pixel wide, V-shaped grid at the region of interest. (C) Black pixels show the identifiable interface, i.e. the presence of gap. The percentage of black pixels (gap) in relation to the length of the adhesive interface was calculated in the software.

Selected B-scans located at half of the distance corresponding to the restoration diameter were used in the analysis for the saucer-shaped restorations (Figure 2). For the wedge-shaped restorations, selected B-scans considered the restoration diameter and the pointed outline of the cavity bottom, which resembled the letter V (Figure 3). In the central part of the restoration, 16 B-scans were averaged (Figures 2A and 3A). On the average image, a grid 20 pixels wide was laid over the area corresponding to the interface, i.e. the region of interest (Figures 2B and 3B). Ten pixels on each side of the interface, running two lines at a time, were used to calculate the background signal as a moving average. A search on the five central pixels on each line was made to identify if the signal intensity at the interface was greater than the averaged background summed to 1.15 times the standard deviation of the background signal, earlier defined as the optimal threshold. If this were the case, the pixel on the first of the two lines was highlighted. When the whole interface had been mapped, the highlighted pixels were used to calculate the percentage gap length, which represents the length of the identifiable interface on the averaged OCT images, in relation to the total length of the interface (Figures 2C and 3C).

In order to identify the reproducibility of the custom-made script, measurements were performed 10 consecutive times in the same B-scans obtained centrally from one restoration. High reproducibility was observed for both NCCL cavity configurations and restorative materials (*I* = 69% with a standard deviation of 2%).

### Thermal aging

After preliminary gap assessment in the microscope and imaging using the SS-OCT, all specimens were submitted to a moderate level of simulated aging by thermocycling for 5000 cycles, in alternating water baths set at temperatures of 5 and 55°C, with a dwell time of 15 seconds each (MW-4 and F32-MV; Julabo GmbH, Seelbach, Germany). The relatively moderate number of cycles was meant to further challenge the adhesive interface beyond the polymerization shrinkage stresses generated by each resin composite, which theoretically differed in magnitude [7]. Subsequently, the specimens were stored in water at room temperature until the subsequent assessment, when new images were obtained using the microscope and the OCT.

### Statistical analysis

The data were analyzed with Statistica for Windows software (StatSoft, Tulsa, USA), with the significance level defined as α = 0.05. Data from marginal and internal gaps were checked for normality using the Shapiro-Wilk test and then analyzed by two-way ANOVA (analysis of variance) to test for the effect of cavity configuration and resin composite. In order to evaluate the influence of TA, paired *t*-tests were performed group by group, comparing the values before and after thermocycling.

## Results

### Microscopy results

The results of the marginal gap assessment are presented in Table 2. The two-way ANOVA revealed that neither the cavity configuration, saucer or wedge, nor the resin composite had a statistically significant effect on marginal gap formation. This was the case before (cavity configuration: *p* = 0.75; resin composite: *p* = 0.77) and after thermocycling (cavity configuration: *p* = 0.20; resin composite: *p* = 0.46). Also, there was no significant interaction between the two factors (before thermocycling: *p* = 0.52; after thermocycling: *p* = 0.89). Thermocycling did not influence the marginal gap formation for any of the four groups (*p* = 0.11 to *p* = 0.84).

**Table 2:** Mean marginal gap formation (expressed as wall-to-wall contraction, %) of the restorations according to cavity configuration and resin composite, observed through light microscopy (512x magnification), and results of paired t-test.

Cavity configuration	Resin composite	Marginal gap before thermocycling	Marginal gap after thermocycling	*P*
Saucer	Bulk-fill composite	0.47 (0.21)	0.46 (0.13)	0.84
	Nanofilled composite	0.53 (0.19)	0.49 (0.15)	0.43
Wedge	Bulk-fill composite	0.49 (0.26)	0.38 (0.17)	0.11
	Nanofilled composite	0.47 (0.1)	0.43 (0.22)	0.58

Standard deviation is given in parentheses.

### OCT results

Table 3 summarizes the results of the internal gap assessments. The two-way ANOVA found no significant effect of the factors cavity configuration and resin composite. This was the case before (cavity configuration: *p* = 0.78; resin composite: *p* = 0.33) and after thermocycling (cavity configuration: *p* = 0.57; resin composite: *p* = 0.15). Also, there was no significant interaction between the two factors (before thermocycling: *p* = 0.27; after thermocycling: *p* = 0.22). Thermocycling did not influence the internal gap formation for any of the four groups (*p* ≤ 0.58).

**Table 3:** Mean internal gap formation (expressed as the length of the gap identified along the adhesive interface, %) of the restorations according to cavity configuration and resin composite, observed through OCT, and results of paired t-test.

Cavity configuration	Resin composite	Internal gap before thermocycling	Internal gap after thermocycling	*P*
Saucer	Bulk-fill composite	52.82 (22.34)	49.95 (20.18)	0.58
	Nanofilled composite	63.48 (19.85)	61.35 (12.57)	0.58
Wedge	Bulk-fill composite	57.05 (8.7)	52.83 (6.78)	0.15
	Nanofilled composite	56.36 (9.68)	53.75 (9.89)	0.45

Standard deviation is given in parentheses.

## Discussion

This *in vitro* study aimed to determine the effect of cavity configuration, resin composite, and thermocycling on the marginal and internal gap formation of NCCL restorations.

Restorations placed in saucer-shaped cavities obtained similar marginal and internal adaptation as restorations placed in wedge-shaped cavities. Thus, the first hypothesis was rejected. Our findings do not comply with earlier stress concentration analyses, which contrastingly have shown more pronounced stress concentration in either saucer-shaped [5] or wedge-shaped [27] cavity geometries. Possible explanations are that the cavities in this study were relatively small, and the volume of composite used in both cavity configurations was comparable. Other than cavity geometry, the size of NCCLs also influences stress concentration at the adhesive interface [5–7, 28, 31]. Higher shrinkage stress and microleakage have been observed in deeper and larger restorations, which most likely is related to the cavity volume rather than its configuration [28, 32]. Such inconsistent results from *in silico* studies make it difficult to estimate the clinical consequences of polymerization stress: a systematic review and meta-analysis of randomized clinical trials [33] did not identify a relationship between cavity shape and retention of resin composite restorations, granted the very low quality of the available clinical evidence. Thus, the influence of cavity configuration on the clinical performance of the resin composite restorations in NCCLs remains unclear [33] and is worthy of further investigation.

In agreement with previous laboratory studies [21, 34], NCCLs restored either with bulk-fill or nanofilled resin composites showed similar marginal and internal gap formation, thus leading to the rejection of the study’s second hypothesis. It is known that internal and marginal adaptation of composite restorations are at large influenced by polymerization shrinkage stresses [16, 21, 23], which in turn are affected by the material’s filler size and content, elastic modulus, and volumetric shrinkage [35]. The resin composites investigated in the present study contain similar amounts of filler (76–78 wt.%) and have similar elastic modulus [12]. The bulk-fill resin composite has a reportedly lower volumetric polymerization shrinkage compared to the nanofilled composite, thanks to its different monomer formulation [36]. According to the manufacturer, the AUDMA (aromatic urethane dimethacrylate) monomer has fewer reactive sites, thus moderating volumetric shrinkage, polymer matrix stiffness, and development of polymerization shrinkage stresses [37]. Although reduced shrinkage stress is assumed for bulk-fill restorations [7, 12], this did not guarantee reduced gap formation. Nevertheless, the clinical success of restorations goes beyond the presence of the marginal gaps, but considers postoperative pain, restoration fracture, or restoration loss. From a material’s perspective, similar clinical performance for bulk-fill and conventional resin composites has been reported in a meta-analysis that included class I and II restorations over a follow-up period up to 72 months [38]. In NCCLs, the same composites employed in this study have shown similar clinical outcomes up to 30 months [15, 39].

Similarly, the adhesive system can influence the quality of the tooth-composite interface [22, 25, 26]. In this study, the self-etching adhesive system Clearfil™ SE Bond, containing the functional monomer 10-methacryloxydecyl phosphate (MDP), was chosen due to its efficacy [40]. Despite this product’s ability to bond well to the dental hard tissues, several studies have reported the presence of gaps in restorations made with this adhesive system and conventional resin composite [25, 41], which is in agreement with our results. Nevertheless, a meta-analysis of long-term randomized clinical trials on NCCLs demonstrated that Clearfil™ SE Bond resulted in good marginal integrity and high (96%) retention rate [42].

Contrary to expectations and previous findings [4, 13], marginal and internal gap formation was not affected by thermocycling, leading to the rejection of the third hypothesis. A possible explanation may be the reduced number of cycles used in this study [43].

Regarding the methods of this study, OCT is a viable imaging tool to evaluate gap formation non-destructively either by assessing 2D images or by following the 3D progression of gaps in real time [17, 21, 22, 24, 26]. When OCT is used, gap formation is identified by a significant increase in signal intensity, i.e. bright clusters on the image result from the presence of air at the adhesive interface. Whilst the development of SS-OCT system has improved sensitivity, increased signal-to-noise ratio and the scanning speed, 3D images provide less details at high speed than 2D images [44]. Thus, averaging 2D images obtained from the OCT improved the reliability of our results. This novel image analysis was accomplished by using a custom-made script for the software MATLAB, which allowed us to detect and quantify the internal gaps on the central portion of the restoration. By increasing the signal-to-noise ratio, it was possible to identify brighter pixels located at the interface, i.e. a gap, right next to its neighboring pixels. The results of the internal gap are expressed in percentage gap length, which indicates the presence of a gap in relation to the total length of the adhesive interface (Figure 2C and 3C). The reproducibility of this image analysis method was high despite the degree of uncertainty inherent to this method.

Another important contribution from our study lies in the assessment of cavity geometries that are closer to the clinical scenario. The assessment of saucer- and wedge-shaped restorations, with cavity margins located partly in enamel and partly in dentine, contrasts with most studies that employ OCT to assess composite restorations. Instead, such studies form their cavities to best suit the OCT imaging method, i.e. cavity geometries that are far from those seen in daily clinical practice, which at best complicate the applicability of their results or question their relevance. By assessing realistic cavity geometries and investigating both internal and marginal adaptation, a thorough assessment of the adhesive interface in NCCLs was achieved. Additionally, by averaging 2D-scans, a greater volume of interest, and consequently better representation, of the adhesive interface could be probed. On the other hand, this work is not without limitations. The presence of small air entrapments within the composite restorations or at the adhesive interfaces that were clearly visible on the light microscope were not always noticeable in the OCT images. Contrarily, voids located directly at, or close to, the adhesive interface were usually misinterpreted as interfacial gaps in the OCT images. Another important limitation of this study concerns the artificially prepared NCCLs, which are not identical to naturally forming NCCLs. During imaging with the OCT, the wedge-shaped cavity walls were at an angle from the incoming light of the OCT device, which compromised light reflection – and consequently image quality – directly from the bottom of these cavities. This optical event adds a large degree of uncertainty to the assessment of wedge-shaped cavities, which showed lower correlation coefficient between observations from the microscope and the OCT. Based on these findings, further development on this image analysis method is warranted to minimize overall measurement uncertainty, especially for wedge-shaped cavity configurations. It is also worth noting that only one brand and two resin composites were evaluated in this study, so the results cannot be generalized.

Within the limitations of this laboratory study, we can conclude that NCCLs saucer- and wedge-shaped restorations presented similar marginal and internal adaptation, regardless of the resin composite employed, and these results were not worsened by thermocycling.

## Data Availability

Data can be made available on request.
